# Effects of hypoxia-inducible factor-prolyl hydroxylase inhibitors *vs*. erythropoiesis-stimulating agents on iron metabolism in non-dialysis-dependent anemic patients with CKD: A network meta-analysis

**DOI:** 10.3389/fendo.2023.1131516

**Published:** 2023-03-16

**Authors:** Junlan Yang, Jie Xing, Xiaodong Zhu, Xiaotong Xie, Lina Wang, Xiaoliang Zhang

**Affiliations:** ^1^ Department of Nephrology, Zhong Da Hospital, Southeast University School of Medicine, Nanjing, Jiangsu, China; ^2^ Department of Epidemiology and Biostatistics, School of Public Health, Southeast University/Hospital, Nanjing, Jiangsu, China

**Keywords:** hypoxia-inducible factor prolyl hydroxylase inhibitor, erythropoiesis-stimulating agent, renal anemia, hepcidin, iron metabolism

## Abstract

**Objective:**

To compare the effects of five hypoxia-inducible factor-prolyl hydroxylase domain inhibitors (HIF-PHIs), two erythropoiesis-stimulating agents (ESAs), and placebo on iron metabolism in renal anemia patients with non-dialysis-dependent chronic kidney disease (NDD-CKD).

**Method:**

Five electronic databases were searched for studies. Randomized controlled clinical trials comparing HIF-PHIs, ESAs, and placebo in NDD-CKD patients were selected. The statistical program used for network meta-analysis was Stata/SE 15.1. The main outcomes were the change in hepcidin and hemoglobin (Hb) levels. The merits of intervention measures were predicted by the surface under the cumulative ranking curve method.

**Results:**

Of 1,589 original titles screened, data were extracted from 15 trials (3,228 participants). All HIF-PHIs and ESAs showed greater Hb level–raising ability than placebo. Among them, desidustat demonstrated the highest probability of increasing Hb (95.6%). Hepcidin [mean deviation (MD) = -43.42, 95%CI: -47.08 to -39.76], ferritin (MD= -48.56, 95%CI: -55.21 to -41.96), and transferrin saturation (MD = -4.73, 95%CI: -5.52 to -3.94) were decreased, while transferrin (MD = 0.09, 95%CI: 0.01 to 0.18) and total iron-binding capacity (MD = 6.34, 95%CI: 5.71 to 6.96) was increased in HIF-PHIs versus those in ESAs. In addition, this study observed heterogeneity in the ability of HIF-PHIs to decrease hepcidin. Compared with darbepoetin, only daprodustat (MD = –49.09, 95% CI: –98.13 to –0.05) could significantly reduce hepcidin levels. Meanwhile, daprodustat also showed the highest hepcidin-lowering efficacy (84.0%), while placebo was the lowest (8.2%).

**Conclusion:**

For NDD-CKD patients, HIF-PHIs could ameliorate functional iron deficiency by promoting iron transport and utilization, which may be achieved by decreasing hepcidin levels. Interestingly, HIF-PHIs had heterogeneous effects on iron metabolism.

**Systematic review registration:**

https://www.crd.york.ac.uk/prospero/display_record.php?RecordID=242777, Identifier CRD42021242777.

## 1 Introduction

Anemia is a common complication in patients with chronic kidney disease (CKD) (1), which is closely associated with an increased risk of CKD progression and mortality. In the last two decades, erythropoiesis-stimulating agents (ESAs) and iron therapy have always been the cornerstone of renal anemia treatment (2). Although ESAs can effectively correct anemia in most patients, there have been concerns about its potential side effects such as increased risks of tumor, cardiovascular events, and cerebrovascular events (3). Therefore, iron supplementation is used to correct iron deficiency and, meanwhile, to minimize the dosage of ESAs. Even for patients with functional iron deficiency, which means that their stored iron is at a relatively high level, intravenous iron therapy has still been performed in the patients undergoing hemodialysis as long as their ferritin level is lower than 500 or even 800 ng/ml (4, 5). However, this kind of practice is liable to put patients at higher risk for infection, allergy, and even iron overload. Hypoxia-inducible factor-prolyl hydroxylase domain inhibitors (HIF-PHIs) are a new class of agents that can inhibit the degradation of the hypoxia-inducible factor (HIF) to promote erythropoiesis by activating the body’s natural physiological response to hypoxia. It stabilizes and promotes the gene expression of the HIF-1 and HIF-2 by inhibiting the activity of prolyl hydroxylase (PH) enzymes (6). These gene actions include increasing the endogenous erythropoietin (EPO) hormone production and regulating iron homeostasis, and the latter ability seems to be their distinct advantage that is not possessed by ESAs. Several HIF-PHIs have been developed in late-stage global clinical development, and some of them have been approved for clinical application (7). Multiple clinical studies have shown that HIF-PHIs can increase hemoglobin (Hb) levels comparable to the ESAs (8, 9), but its effect on iron metabolism has not been definitively elucidated.

Iron transport disorder is a common cause of refractory anemia in CKD patients (10). CKD patients are subject to be in a chronic inflammatory state, which promotes the abnormal increase of hepcidin levels, resulting in abnormal iron storage and the disorder of the transport of iron. In hyperinflammatory states, this condition even becomes a leading factor in refractory renal anemia. Several large randomized controlled trials (RCTs) have found that HIF-PHIs had a markedly significant effect on iron regulation in the treatment of anemia, mainly manifested as decreased hepcidin, transferrin saturation (TSAT) and ferritin (11), decreased or unchanged serum iron (9), and increased transferrin and total iron-binding capacity (TIBC). These results suggested that HIF-PHIs can improve iron transport and iron utilization in patients. However, it is generally accepted that NDD-CKD populations have a lower risk of chronic inflammatory state compared with dialysis-dependent populations. Some results from several latest RCTs have demonstrated that HIF-PHIs could improve iron metabolism in NDD-CKD populations (12–14). However, the lack of head-to-head comparisons makes it impossible to generalize any conclusions regarding the differences of iron-modulating efficacy between HIF-PHIs and ESAs or among different types of HIF-PHIs. Thus, this study is done to explore whether HIF-PHIs still have therapeutic advantages in regulating iron metabolism in a lower inflammatory state, evaluate the differences in iron-regulating efficacy between different HIF-PHIs, and finally develop evidence-based strategy for the treatment of CKD anemia.

## 2 Research methods

The study protocol is available on the International Prospective Register of Systematic Reviews (PROSPERO) with a registration number of CRD42021242777. Three investigators (Jie Xing, Xiaodong Zhu, and Xiaotong Xie) independently performed study selection and quality assessment according to the study protocol. Any disagreements were resolved by consultation with another investigator (Lina Wang).

### 2.1 Inclusion and exclusion criteria

#### 2.1.1 Study type

Only randomized controlled trials can be included in this study.

#### 2.1.2 Subjects

Only patients with renal anemia without dialysis older than 18 years were included in this study.

#### 2.1.3 Intervention measures

The experimental group received any one of the interventions: roxadustat, daprodustat, molidustat, desidustat, enarodustat, and vadadustat, as the control group received any one of the interventions epoetin, darbepoetin, and placebo. The other two groups received the same intervention measures. The treatment time is more than 4 weeks.

#### 2.1.4 Outcome indicators

The main outcome indexes included the changes in hepcidin (ng/ml) and Hb (g/dl) before and after treatment. Secondary outcome indexes included the changes in ferritin (µg/l), serum iron (μmol/l), transferrin (g/l), TIBC (μmol/l) and TSAT(%) before and after treatment.

#### 2.1.5 Exclusion criteria

Trials using the same data were included in this study only as one trial. (1) Data duplication or has been included in other tests. (2) The patient uses a combination of multiple intervention drugs or the intervention group and the control group are different doses of the same drug. (3) The patient as anemia caused by other reasons than renal anemia. (4) The article does not report the patient’s changes in hepcidin and Hb before and after treatment. (5) Publications are neither in Chinese nor in English. (6) Publications are letters, comments, conference abstracts, editorials, and case reports or case series.

### 2.2 Literature retrieval strategy

**Figure 1:**
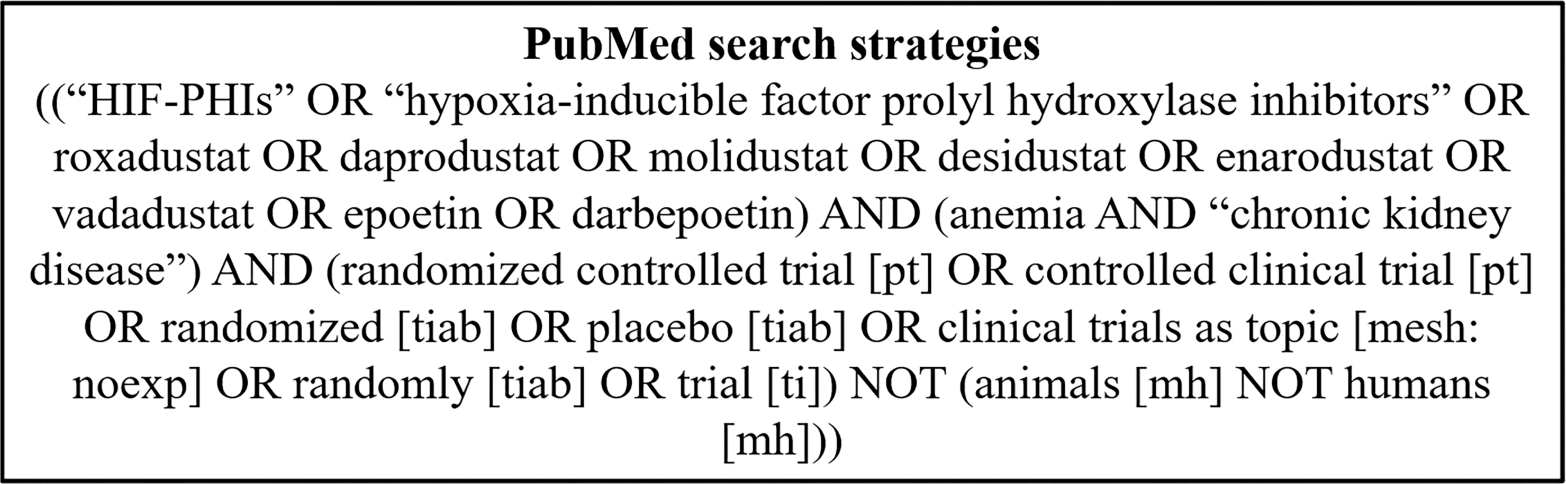
Examples of PubMed search strategies.

The Cochrane library, PubMed (**Figure 1**), Web of Science, Chinese journal full-text database, and WANGFANG DATA were used for search in documents and journals ranging from January 1946 to March 2022. The publications, with respect to the randomized controlled clinical trial comparing HIF-PHIs, ESAs, and placebo in renal anemia patients with NDD-CKD, were selected. The main outcomes included the change in hepcidin and Hb levels.

### 2.3 Study selection and outcomes

We extracted information using a predesigned data collection forms, which included literature name, first author, publication time and journal, sample size of each group, patients’ average age, CKD status in each group, interventions and follow-up time, the key elements of evaluating the quality of the included study and outcome indicators concerned.

### 2.4 Risk assessment of literature bias

The revised Cochrane risk-of-bias tool for randomized trials (RoB 2) was used to evaluate the quality of the included literature. RoB 2 is a tool that has six evaluation items, including the risk of bias arising from the randomization process, the risk of bias due to deviations from the intended interventions (the effect of assignment to intervention), missing outcome data, the measurement of the outcome, the risk of bias in the measurement of the outcome, the risk of bias in the selection of the reported result, and the overall risk of bias. The evaluation results are divided into three levels: high risk of bias, unclear risk of bias, and low risk of bias.

### 2.5 Statistical analysis

This study used Stata/SE 15.1 to conduct network meta-analysis. All statistical analyses were performed based on the frequentist framework. Network maps were used to show the interactions among the treatment comparisons. In this study, two methods, global inconsistency and the node-splitting method, were used to evaluate inconsistency. Global inconsistency is a test to compute the inconsistency between the treatment comparison of all studies, while the node-splitting method is a local inconsistency test, which examines each treatment individually. If two inconsistency tests are *P* > 0.05, it means that inconsistency was found to be not significant in both global and local tests. For star networks without closed loops (networks where all treatments are compared with a common treatment but not between themselves), no inconsistency test was conducted, and they are only evaluated conceptually for all indirect comparisons to derive valid network meta-analysis estimates. We calculated mean deviation (MD) and 95% confidence interval (CI) to analyze continuous variables. When the 95% CI of MD contains zero, it indicates that the disagreement between interventions is considered not to be significant. The surface under the cumulative ranking curve (SUCRA) was used to sort the treatments in rank; the higher SUCRA probabilities, the superior the treatment effect. Publication bias was assessed by a visual inspection of the comparison-adjusted funnel plot.

## 3 Results

### 3.1 Literature screening process and results

**Figure 2:**
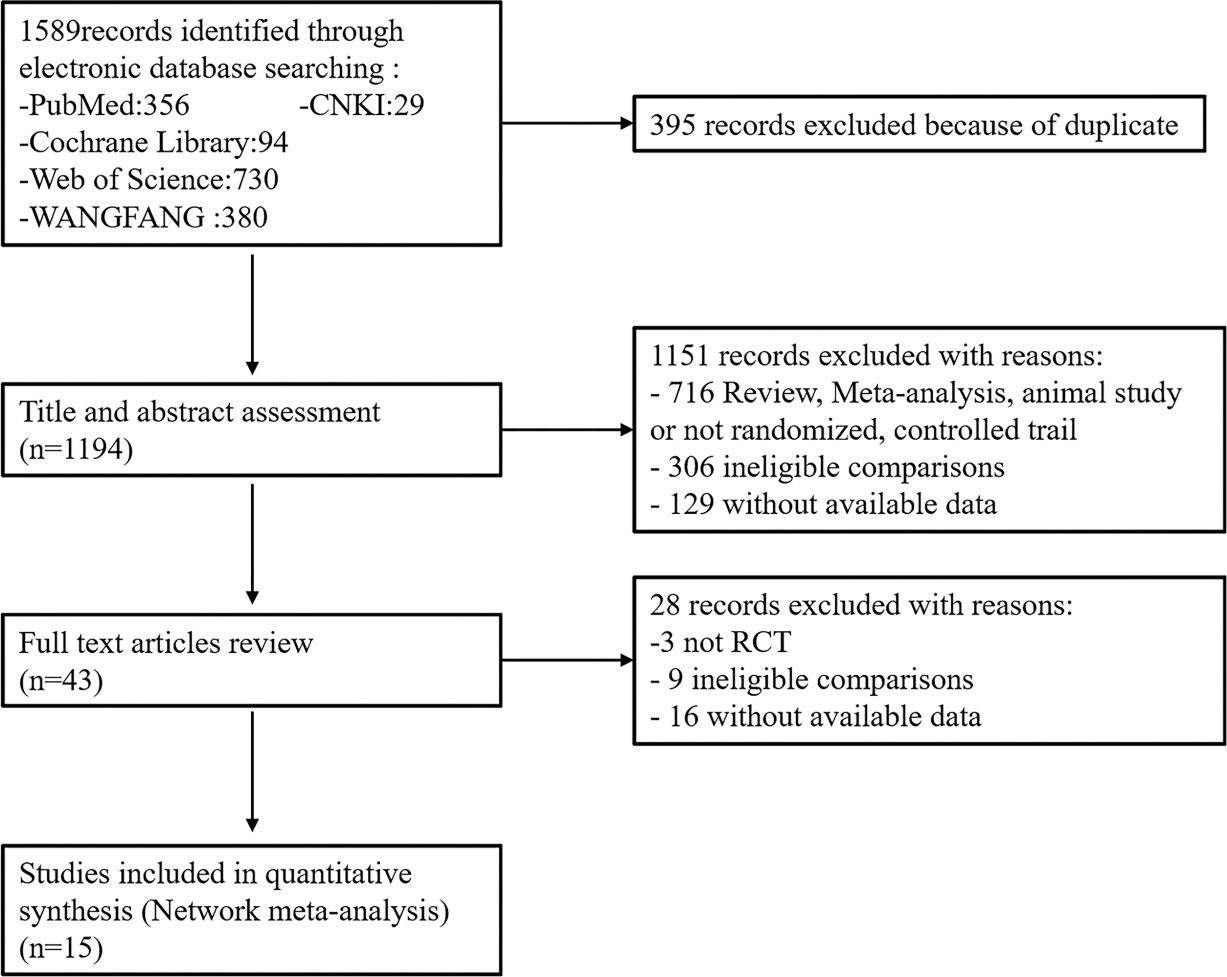
Flow diagram of study selection process.

This study followed the Preferred Reporting Items for Systematic Reviews and Meta-analyses (PRISMA) guideline (Supplementary PRISMA network meta-analysis (NMA) Checklist). A total of 1,589 related articles were retrieved based on the preliminary search strategy. There were 15 trials that met the predefined inclusion criteria and were included in our network meta-analysis (**Figure 2**). The 15 trials included 3,228 patients (**Table 1**).

**Table 1:** Characteristics of included studies.

Study (year)	Sample size (Intervention/control)	CKD5 (Intervention/control, %)	eGFR (Intervention/control, ml/min/1.73 m ^2^)	CKD status	Mean age (Intervention/control, years)	Follow-up	Trial drugs	Control	Outcomes
Shutov E. (12)	391/203	26.1/30	16.5 ± 10.2/17.2 ± 11.7	CKD3-5	60.6 ± 13.5/61.7 ± 13.8	52W	Roxadustat	Placebo	abdg
Akizawa T. (14)	107/109	48.5/50	18.6 ± 10.1/17.3 ± 8.3	CKD3-5	70.4 ± 9.1/68.9 ± 9.1	24w	Enarodustat	Darbepoetin	abcdfg
Coyne, D. W. (13)	616/306	20.1/24.8	21.9 ± 11.5/22.4 ± 11.4	CKD3-5	64.9 ± 12.6/64.8 ± 13.2	52w	Roxadustat	Placebo	abcdfg
Nangaku M. (15)	37/14	54/29	1.78 ± 10.3/22.0 ± 9.8	CKD3-5	69.8 ± 11.7/71.4 ± 11.6	6w	Vadadustat	Placebo	abcdfg
Parmar DV. (16)	87/30	0/0	-/-	CKD1-4	48.5 ± 12.2/46.9 ± 12.7	6w	Desidustat	Placebo	abcef
Holdstock L. I (17)	74/37	32/44	20.6 ± 10.45/19.2 ± 11.3	CKD3-5	66.4 ± 12.8/65.4 ± 13.6	24w	Daprodustat	Epoetin	abdfg
Holdstock L. II (17)	74/37	32/45	20.6 ± 10.45/19.2 ± 11.4	CKD3-5	66.4 ± 12.8/65.4 ± 13.6	24w	Daprodustat	Darbepoetin	abdfg
Chen, N. (18)	101/51	18/47	16.5 ± 8/14.5 ± 7.6	CKD3-5	54.7 ± 13.3/53.2 ± 13.1	9w	Roxadustat	Placebo	abcdef
Akizawa, T. I (19)	71/23	40.9/47.8	18.1 ± 8.7/16 ± 6.4	CKD3-5	68.6 ± 20.0/65.3 ± 14.7	6w	Enarodustat	Placebo	abdfg
Akizawa, T. II (19)	79/24	40.9/47.8	18.1 ± 8.7/16 ± 6.4	CKD3-5	68.0 ± 10.7/66.2 ± 11.9	6w	Enarodustat	Placebo	abdfg
Akizawa, T. (20)	80/27	50/48	16.3 ± 7.8/16.3 ± 8.5	CKD3-5	64.4 ± 9.6/61.9 ± 10.6	6w	Roxadustat	Placebo	abdefg
Martin, E. R. (2017) (21)	72/19	0/0	24.4 ± 11.1/25.2 ± 11.1	CKD3-4	65.7 ± 9.7/64.9 ± 10.0	6w	Vadadustat	Placebo	abdf
Chen, N. (22)	61/30	37.7/30	19.4 ± 9.5/23 ± 13.4	CKD3-5	48.9 ± 13.8/51.4 ± 11.9	8w	Roxadustat	Placebo	abdefg
Pergola, P. E. (3)	138/72	12.3/16.7	25.2 ± 10.4/25 ± 11.7	CKD3-5	66.6 ± 10.0/65.9 ± 12.3	20w	Vadadustat	Placebo	abcdfg
Holdstock, L. (23)	54/18	25.9/33	24.1 ± 10.9/23.2 ± 11.5	CKD3-5	68.4 ± 11.5/69.2 ± 11.0	4w	Daprodustat	Placebo	abdefg
Brigandi, R. A. (24)	61/9	-/-	23.6 ± 10.1/24.3 ± 10.6	CKD3-5	58.4 ± 13.9/54.7 ± 17.3	4w	Daprodustat	Placebo	abdfg
Besarab, A. (25)	88/28	0/0	34.3 ± 12.7/31.4 ± 12.4	CKD3-4	64.0 ± 18.0/68.6 ± 10.4	4w	Roxadustat	Placebo	abcdfg

a: hepcidin, b: hemoglobin (Hb), c: serum iron, d: ferritin, e: transferrin, f: total iron-binding capacity (TIBC), g: transferrin Saturation (TSAT).

### 3.2 Basic information included in the study

The 15 trials (3, 12–25) included a total of 3,228 patients (**Table 1**). The two trials (17, 19) were split into two separate studies for analysis because they used two interventions to compare with a control group. There were five HIF-PHIs involved including roxadustat (n = 6), enarodustat (n = 3), vadadustat (n = 3), desidustat (n = 1), daprodustat (n = 4), and two ESAs involved including darbepoetin (n = 2) and epoetin (n = 1). Among them, hepcidin and Hb levels were reported in 15 trials (17 studies), 7 trials (7 studies) reported serum iron, and 14 trials (16 studies) reported ferritin. The dosing schedule, iron usage, blinding method, and registration number of the included studies are shown in **Table S1**.

### 3.3 Risk assessment of literature bias

**Figure 3:**
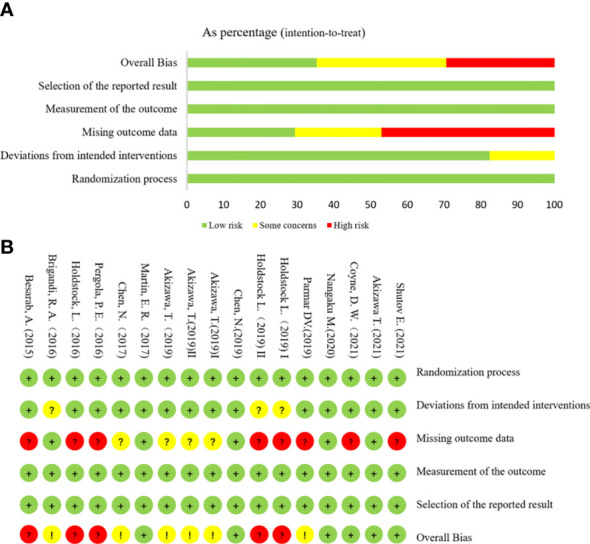
Risk assessment of literature bias. **(A)** The quality assessment for each bias risk item is presented as a percentage. **(B)** Quality assessment of each bias risk item for each study. Each color represents a difference in the risk of bias: green represents low risk of bias, yellow represents unclear risk, and red represents high risk of bias.

The risk assessment of the literature bias of 15 trials (17 studies) is shown in **Figure 3**. All evaluation items of the four studies were at a low risk of bias. Three studies could not determine their risk of bias due to deviations from the intended interventions because carers and people delivering the interventions are aware of participants’ assigned intervention during the trial A total of 12 studies could not be judged as low bias risk because they may miss outcome data.

### 3.4 Network map

**Figure 4:**
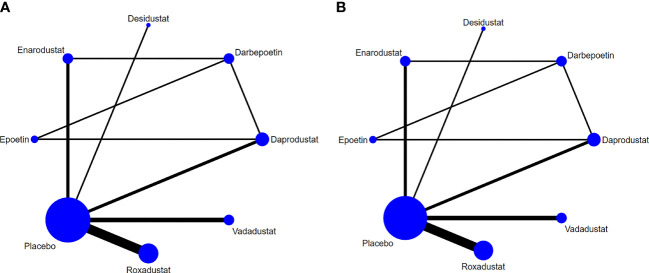
Network graphs of all the drug agents included in the study. The width of the lines is proportional to the number of trials comparing each pair of treatments. The sizes of the circles are proportional to the number of trials using this intervention. **(A)** hepcidin, **(B)** Hb.

The relationship between eight various intervention measures reporting changes in hepcidin and Hb before and after treatment is shown in **Figure 4**. The connection means that the two interventions are directly compared. This study involved a comparison of eight intervention measures: roxadustat, enarodustat, vadadustat, desidustat, daprodustat, darbepoetin, epoetin, and placebo. Of all trials, roxadustat and placebo were compared in six trials, which is more than any other pairs. In the experimental group, the largest number of patients used was 1,337 for roxadustat.

### 3.5 League table

**Figure 5:**
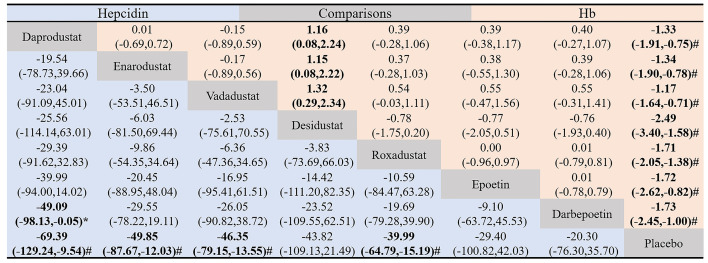
Pairwise comparison of the hepcidin-lowering and hemoglobin-raising effectiveness of the eight drugs included in the study. Data in each grid represent MD and 95% CI. Column-defining drugs are compared to the row-defining drugs. The lower triangle (blue) shows pairwise comparisons of the effectiveness of hepcidin-lowering among the eight interventions, and the upper triangle (red) shows pairwise comparisons of the effectiveness of hemoglobin-raising among the eight interventions. The Difference between the two interventions was considered statistically significant when the 95% CI for MD did not include 0. #, significant difference compared to placebo. *, significant difference compared to ESAs.

The league table shows pairwise comparisons of the effectiveness of hepcidin lowering and Hb raising among the eight interventions (**Figure 5**) with no inconsistency between groups (hepcidin: *P* =0.18, Hb: *P* = 0.97). The results of the inconsistency and consistency tests are presented in **Tables S2** and **S3**. Compared with placebo, daprodustat (MD = -69.39, 95% CI: -129.24 to -9.54), enarodustat (MD = -49.85, 95% CI: -87.67 to -12.03), vadadustat (MD = -46.35, 95% CI: -79.15 to -13.55), and roxadustat (MD = -39.99, 95% CI: -64.79 to -15.19) could significantly reduce the level of hepcidin. Enarodustat also significantly reduced hepcidin levels compared with darbepoetin (MD = -49.09, 95% CI: -98.13 to -0.05). Although the results of other HIF-PHIs compared with placebo and ESAs were not statistically significant, they all suggested that HIF-PHIs may have a stronger ability to reduce hepcidin. All HIF-PHIs and ESAs showed a greater ability to increase Hb levels than placebo, and there was no significant difference between HIF-PHIs and ESAs.

### 3.6 Surface under the cumulative ranking curve

**Figure 6:**
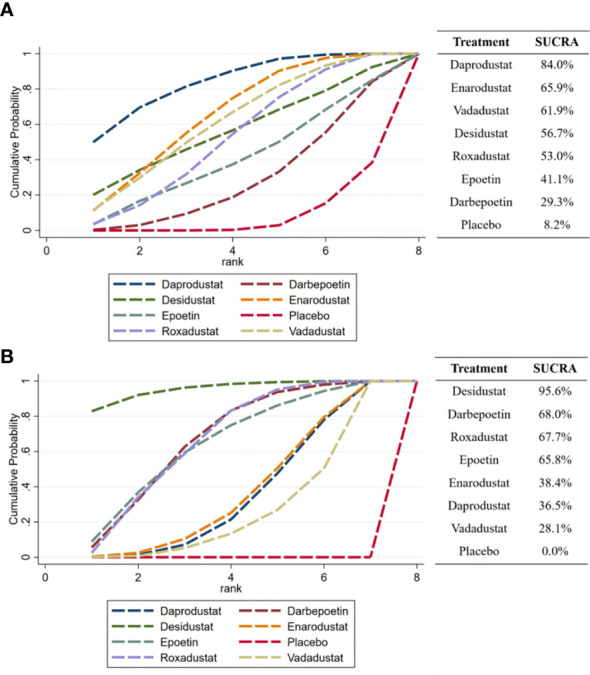
SUCRA ranking of the effectiveness of each drug in reducing hepcidin and increase Hb. **(A)** hepcidin, **(B)** Hb.

The SUCRA chart gives a ranking of the effectiveness of each intervention in reducing hepcidin (**Figure 6A**). The higher the percentage is, the more hepcidin is reduced. The result that could be obtained from the SUCRA was that daprodustat ranked first (84.0%) and placebo ranked last (8.2%). All HIF-PHIs (daprodustat, enarodustat, vadadustat, desidustat, and roxadustat) included in the comparison were more effective at lowering hepcidin than ESAs and placebo. **Figure 6B** reports the effectiveness ranking of various drugs to increase Hb levels, and it can be found that desidustat ranked first (95.6%), which was higher than darbepoetin (68.0%) and epoetin (65.8%).

### 3.7 Effects of hypoxia-inducible factor-prolyl hydroxylase domain inhibitors or erythropoiesis-stimulating agents on the regulation of iron metabolism

**Figure 7:**
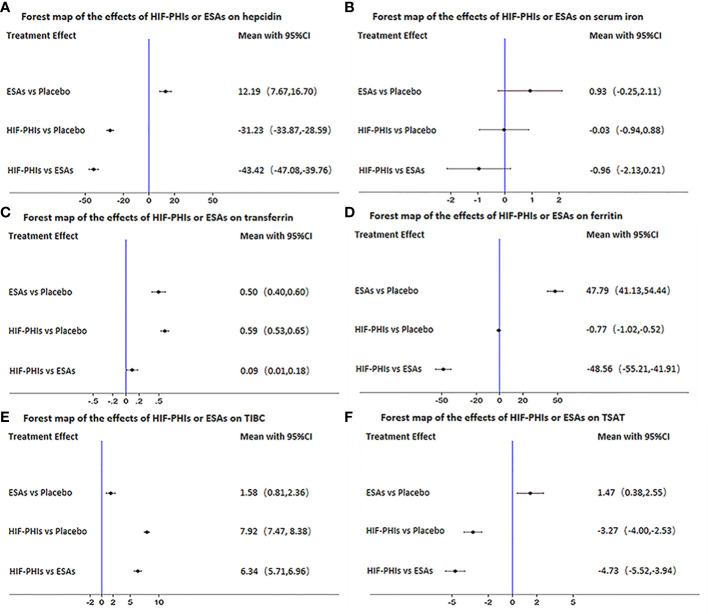
Forest map of the effects of three drugs on the regulation of iron metabolism. **(A)** hepcidin, **(B)** serum iron, **(C)** transferrin, **(D)** ferritin, **(E)** TIBC, **(F)** TSAT.

Of the included 15 trials (17 studies), 15 trials (17 studies) provided hepcidin, 7 trials (7 studies) provided serum iron, 5 trials (5 studies) provided transferrin, 14 trials (16 studies) provided ferritin, 14 trials (16 studies) provided TIBC, and 11 trials (13 studies) provided TSAT (**Figure 7**). The network of the indices of iron metabolism is presented in **Figure S1**. HIF-PHIs could significantly decrease hepcidin levels in comparison with placebo (MD = -31.23, 95%CI: -33.87 to -28.59) and ESAs (MD=-43.42, 95%CI: -47.08 to -39.76), while ESAs could significantly raise hepcidin levels than placebo (MD = 12.19, 95%CI: 7.67 to 16.70) (**Figure 7A**). There was no statistical difference in the effect of all comparisons on serum iron (**Figure 7B**). Both HIF-PHIs (MD = 0.59, 95%CI: 0.53 to 0.65) and ESAs (MD = 0.50, 95%CI: 0.40 to 0.60) increased transferrin more than placebo. HIF-PHIs could significantly increase transferrin levels in comparison with ESAs (MD = 0.09, 95%CI: 0.01 to 0.18) (**Figure 7C**). HIF-PHIs could significantly decrease ferritin levels in comparison with placebo (MD=-0.77, 95%CI: -1.02 to- 0.52) and ESAs (MD=-48.56, 95%CI: -55.21 to -41.91), while ESAs could significantly raise ferritin levels than placebo (MD = 47.79, 95%CI: 41.13 to 54.44) (**Figure 7D**). The ability of HIF-PHIs to increase TIBC was significantly stronger than placebo (MD = 7.92, 95%CI: 7.47 to 8.38) and ESAs (MD = 6.34, 95%CI: 5.71 to 6.96) (**Figure 7E**). HIF-PHIs significantly reduced TSAT compared with placebo (MD=-3.27, 95%CI: -4.00 to -2.53) and ESAs (MD = -4.73, 95%CI: -5.52 to -3.94), while ESAs could significantly raise TSAT than placebo (MD = 1.47, 95%CI: 0.38 to 2.55) (**Figure 7F**).

### 3.8 Publication bias test

**Figure 8:**
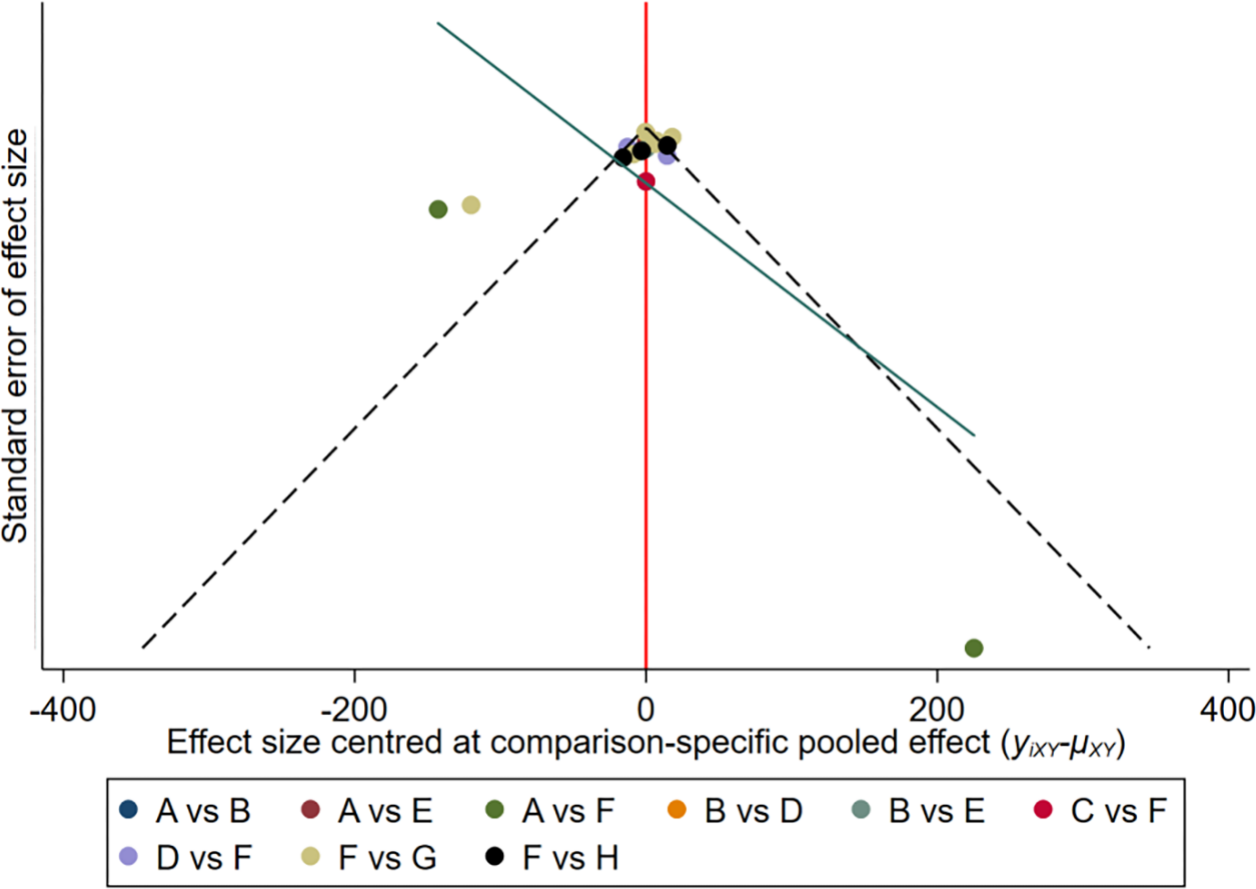
The comparison-adjusted’ funnel plot of the changes in hepcidin of each intervention. A: Daprodustat; B: Darbepoetin; C: Desidustat; D: Enarodustat; E: Epoetin; F: Placebo; G: Roxadustat; H: Vadadustat.

After drawing the comparison-adjusted funnel plot of the changes in the hepcidin of each intervention (**Figure 8**), it can be seen that most of the study results are clustered around the red line (null hypothesis), representing that the study-specific effect sizes do not differ from the respective comparison-specific pooled effect estimates, and the overall risk of bias is small. However, there are still small studies that tend to overstate the hepcidin-lowering ability of drugs, and publication bias remains.

## 4 Discussion

This study is the largest network meta-analysis to compare the effects of five HIF-PHIs, two ESAs, and placebo on correcting anemia and iron dysregulation. The ability of all five HIF-PHIs included in this studyto correct anemia is not inferior to ESAs. In addition, desidustat exhibited the strongest Hb-increasing ability. When analyzing the regulatory effects of different kinds of drugs on iron metabolism, this study took five HIF-PHIs as one group and two ESAs as the other group, which made the conclusion more extrapolative. The results of this study showed that, compared with placebo and ESAs, HIF-PHIs could significantly decrease hepcidin, TSAT, and ferritin and increase transferrin and TIBC but did not alter serum iron. This network meta-analysis also observed heterogeneity in the ability of different HIF-PHIs to decrease hepcidin. After calculating the effectiveness of each drug to decrease hepcidin, this study found that daprodustat had the highest hepcidin-lowering efficacy.

ESAs are currently the most widely used drugs for the treatment of renal anemia. Even without considering their potential cardiovascular events and cerebrovascular event risks, some patients who have been in a state of inflammation for a long time would not respond to ESAs, known as ESA hyporesponse (26), making their ability to correct anemia non-ideal. Oxygen-sensing and adaptation mechanisms are among the most important mechanisms of life. Three decades ago, it was discovered that the HIF is a key factor for the body to increase EPO production and correct anemia during hypoxia (27). When oxygen levels drop, HIF-α subunits accumulate and dimerize with the HIF-β to form functional transcription factors HIF-1 and HIF-2, which can directly regulate the expression of the EPO gene and other genes involved in encoding iron absorption–related proteins, thereby correcting anemia. In the presence of sufficient oxygen, the HIF is hydroxylated by HIF-PH enzymes and degraded by ubiquitination (28). HIF-PHIs are a new kind of anemia treatment agent that inhibits the degradation of the HIF by simulating the natural physiological response of the body to hypoxia. A previous network meta-analysis (2,768 patients) (29) reported that HIF-PHIs have an anemia treatment effect similar to ESAs and safer to some extent. However, the accuracy and clinical value of the results of that study were limited because of the small sample size and the lack of direct head-to-head comparisons between different HIF-PHIs. This network meta-analysis enrolled four newly published large studies (these four studies included 1,783 NDD-CKD renal anemia patients), allowing for a greater accuracy of the results and a further extrapolation of the conclusions. In this study, the result showed that the five HIF-PHIs included were non-inferior to ESAs in their ability to correct anemia, and desidustat even showed a stronger ability to increase Hb levels than ESAs.

Iron transport disorder is very common in CKD patients and an important cause of refractory renal anemia, which can be manifested as decreased serum iron and transferrin (30) and increased ferritin after adequate iron supplementation but still low levels of TSAT (31).The iron dysregulation in CKD patients is associated with abnormally increased hepcidin under the inflammatory state (32). Hepcidin is encoded by the HAMP gene, which is located on chromosome 19 q13, and is mainly produced and secreted by hepatocytes. The larger precursor protein produced by hepatocytes undergoes continuous cleavages to finally form a 25-amino acid peptide hormone, which is the main bioactive and predominant form of hepcidin. Hepcidin can modulate iron metabolism by a negative feedback mechanism and is a key hormone for the body to maintain iron homeostasis (33). The decrease of the hepcidin level promotes the release of more iron into the blood from hepatocytes and macrophages, as well as increased intestinal iron uptake, ultimately increasing the iron level in the circulation (30). Can HIF-PHIs ameliorate iron metabolism? Previous meta-analysis has initially observed that HIF-PHIs can decrease hepcidin (34), but there is the lack of exploration of potential differences between different HIF-PHIs. This network meta-analysis found that HIF-PHIs can correct anemia without changing serum iron levels because HIF-PHIs could decrease hepcidin, thereby promoting the transport and utilization of iron. In addition, HIF-PHIs can directly induce hepatic and renal EPO expression and increase the transcription of several iron transport and metabolism genes and finally promote erythropoiesis by improving iron transport (35). This study found that HIF-PHIs had different regulatory effects on iron metabolism, among which daprodustat had the highest hepcidin-lowering efficacy, and enarodustat was second only to it. HIF-PHIs have heterogeneous effects on iron metabolism, possibly because of their different pharmacokinetic profiles and potential drug–drug interactions (7).

The regulatory effects of HIF-PHIs on iron metabolism were also presented in the changes of other iron-related indicators. Iron dysregulation can be divided into absolute and relative iron deficiency according to the level of stored iron (25). Ferritin is an important indicator of the iron storage status of the body. Functional iron deficiency, characterized by elevated ferritin levels that are not available for erythropoiesis, is one cause of ESA hyporesponse in patients with renal anemia (36). This network meta-analysis found that HIF-PHIs decreased ferritin compared to ESAs. However, this did not mean that HIF-PHIs aggravated iron deficiency. Rather, because it promoted the utilization of stored iron (37), the reduction of ferritin by HIF-PHIs was a performance of improved iron transport and bioavailability.

The main function of transferrin is to bind and transport iron in the circulation. TIBC is determined by the level of transferrin, and their increases both imply improved iron transport. TSAT is the ratio of serum iron to TIBC, generally considered as an important biochemical marker of the iron level in the circulation and reflecting iron availability for erythropoiesis. Thus, a decrease in TSAT represents iron deficiency (31). Interestingly, unlike previous studies, this network meta-analysis found that HIF-PHIs could significantly increase transferrin and TIBC and decrease TSAT, without changing serum iron levels, which was a good signal (38). HIF-PHIs increased iron utilization in the bone marrow and failed to increase serum iron levels, but, at the same time, increased transferrin led to elevated TIBC. Therefore, as the ratio of serum iron to TIBC, the reduction of TSAT in this case did not mean functional iron deficiency, but it was a sign of improved iron transport. The said dynamic perspective for analyzing the causation of TSAT changes was obviously different from the traditional static perspective for evaluating iron metabolism. On the contrary, ESAs need to be combined with iron therapy to increase serum iron levels and lead to elevated TSAT, and the elevation of the TSAT in this context did not imply an improvement in iron metabolism. Therefore, ESAs could not improve iron transport but increase the risk of iron overload due to the need for iron supplementation. At the same time, iron supplementation feedback increased the production of hepcidin by hepatocytes, which easily led to the vicious circle of iron dysregulation. In conclusion, in the application of HIF-PHIs, due to its multifaceted role in ameliorating iron transport, the evaluation of iron-related indicators should break through the original static perspective, and it is necessary to dynamically observe and evaluate iron metabolism and transport in patients.

This network meta-analysis had several limitations: firstly, some of the included trials did not use the double-blind method, but, considering that the included outcomes of this study have a unified standard and are not easy to be changed by subjective factors, non-double-blind trials are not excluded. Secondly, the follow-up time between the included trials was quite different; thus, this study could not accurately observe the iron metabolism of patients in different administration stages. However, considering that most HIF-PHIs have not yet been approved for clinical use, this study still included some phase II trials with shorter dosing time. Finally, there were differences in the CKD status of the subjects in the included trials, which might make their iron metabolism status different. However, after calculation, it was found that this difference did not meet the statistical criteria for heterogeneity; thus, this study did not adjust the inclusion and exclusion criteria.

In conclusion, the results of this study suggested that HIF-PHIs should be the first choice in the treatment of NDD-CKD patients with functional iron deficiency. In addition, the potential heterogeneity of HIF-PHIs to correct iron metabolism should be fully considered when formulating a treatment plan.

## Data availability statement

The original contributions presented in the study are included in the article/**Supplementary Material**. Further inquiries can be directed to the corresponding author.

## Author contributions

XLZ and LW designed this study. JX, XDZ and XX searched the literature. JY analyzed the data and wrote the manuscript. All authors contributed to the article and approved the submitted version.
