# Supplementary material for: Effects of hypoxia-inducible factor-prolyl hydroxylase inhibitors vs. erythropoiesis-stimulating agents on iron metabolism in non-dialysis-dependent anemic patients with CKD: A network meta-analysis
Source: Front Endocrinol (Lausanne). 2023 Mar 16;14:1131516. doi: 10.3389/fendo.2023.1131516 (PMC10060950; doi:10.3389/fendo.2023.1131516)
Supplement: Supplementary file 1 [file DataSheet_1.docx]

**Effects of hypoxia-inducible factor-prolyl hydroxylase inhibitors vs erythropoiesis-stimulating agents on iron metabolism in** **non-dialysis-dependent anemic patients with CKD: a network meta-analysis**

**Junlan Yang^1^, Jie Xing^1^, Xiaodong Zhu^1^, Xiaotong Xie^1^, Lina Wang^2^, Xiaoliang Zhang*^1^**

*** Correspondence:**Xiaoliang Zhang: Department of Nephrology, Zhong Da Hospital, Southeast University School of Medicine, 87 Ding Jiaqiao Rd., Nanjing, Jiangsu, 210009, China. Tel: +86 13852290358. E-mail: tonyxlz@163.com

**Table S1. Treatment plans and blinding method of included studies.**

| Study (year) | trial drugs | controrl | Sample size (Intervention/control) | Follow-up | Iron usage | Dosing schedule | Blinded | Registration number |
| --- | --- | --- | --- | --- | --- | --- | --- | --- |
| Shutov E.(2021) | Roxadustat | Placebo | 391/203 | 52W | Oral and intravenous iron was allowed | Participants received roxadustat according to the tiered weight-based approach, with starting doses of 70 mg given thrice weekly (TIW) to participants weighing up to 70 kg and 100 mg given TIW to participants weighing more than 70 kg. | double-blind | NCT01887600 |
| Akizawa T.(2021) | Enarodustat | Darbepoetin | 107/109 | 24w | Oral and intravenous iron was allowed | Subjects assigned to the enarodustat arm received 2 mg/d as the initial dose. In the DA arm, ESA-naïve patients received 30 μg/2 wks as the initial dose whereas dose and frequency administered to ESA-treated patients were determined based on their prior ESA regimen | double-blind | JapicCTI-183870 |
| Coyne, D. W.（2021） | Roxadustat | Placebo | 616/306 | 52w | IV iron was not allowed | Patients weighing 45 to <70 kg received 70 mg roxadustat or placebo, and those weighing ≥70 kg received 100 mg thrice weekly | double-blind | NCT01750190 |
| Nangaku M.(2020) | Vadadustat | Placebo | 37/14 | 6w | IV iron was not allowed | 150, 300 or 600 mg, QD | double-blind | NCT03054337 |
| Parmar DV.(2019) | Desidustat | Placebo | 87/30 | 6w | - | 100, 150 or 200 mg, every alternate day | double-blind | CTRI/2017/05/008534 |
| Holdstock L.（2019）I | Daprodustat | Epoetin | 74/37 | 24w | Oral and intravenous iron was allowed | 1, 2 or 4 mg ,QD;2 mg,QD | single-blind (subjects) | NCT01977573 |
| Holdstock L.（2019）II | Daprodustat | Darbepoetin | 74/37 | 24w | Oral and intravenous iron was allowed | 1, 2 or 4 mg ,QD;2 mg,QD | single-blind (subjects) | NCT01977573 |
| Chen, N.(2019) | Roxadustat | Placebo | 101/51 | 9w | parenteral iron was withheld | Patients initiated roxadustat according to a weight-based starting dose of 70 mg (in patients weighing 40 to <60 kg) or 100 mg (in patients weighing ≥60 kg), TIW | double-blind | NCT02652819 |
| Akizawa, T.(2019)I | Enarodustat | Placebo | 71/23 | 6w | intravenous iron preparations were prohibited and oral iron preparations were permitted only if they had been used before Scr Visit 1; however, a change in dose regimen was prohibited | 2, 4 or 6 mg, QD | double-blind | JapicCTI-152881 |
| Akizawa, T.(2019)II | Enarodustat | Placebo | 79/24 | 6w | intravenous iron preparations were prohibited and oral iron preparations were permitted only if they had been used before Scr Visit 1; however, a change in dose regimen was prohibited | 2, 4 or 6 mg, QD | double-blind | JapicCTI-152881 |
| Akizawa, T.（2019） | Roxadustat | Placebo | 80/27 | 6w | The concomitant use of oral iron was allowed; intravenous iron was permitted only if TSAT was < 5% and serum ferritin was < 30 ng/mL | 50, 70 or 100 mg, TIW | double-blind | NCT01964196 |
| Martin, E. R.（2017） | Vadadustat | Placebo | 72/19 | 6w | All subjects received low-dose supplemental oral iron (50 mg daily) | 240, 370, 500, or 630 mg ,QD | double-blind | NCT01381094 |
| Chen, N.（2017） | Roxadustat | Placebo | 61/30 | 8w | allowed supplementation with oral iron at investigator discretion | low (1.1–1.75 mg/kg) or high (1.50–2.25 mg/kg), TIW | double-blind | NCT01599507 |
| Pergola, P. E.（2016） | Vadadustat | Placebo | 138/72 | 20w | Oral iron supplementation was permitted throughout the study to maintain ferritin levels between 50 and 300 ng/ml; i.v. iron was only allowed for patients who were intolerant of oral iron | Once-daily vadadustat was initiated at 450 mg, and titrated by 1 tablet (150 mg) according to Hb response (maximum of 600 mg and minimum of 150 mg) | double-blind | NCT01906489 |
| Holdstock, L.（2016） | Daprodustat | Placebo | 54/18 | 4w | - | 0.5 mg、2 mg or 5 mg, QD | double-blind | NCT01587898 |
| Brigandi, R. A.（2016） | Daprodustat | Placebo | 61/9 | 4w | - | 10、25、 50 or 100 mg，QD | single-blind (subjects) | - |
| Besarab, A.（2015） | Roxadustat | Placebo | 88/28 | 4w | If the subject was on oral iron at BL during enrollment, they had to remain on a stable dose of oral iron throughout the study | 0.7、1.0、1.5 and 2.0 mg/kg ,BIW or TIW | single-blind (subjects) | NCT00761657 |

| **overall inconsistency** | | | | |
| --- | --- | --- | --- | --- |
| **comparisons** | **Consistency Model** | | **inconsistency model** | |
|  | **MD（95%CI）** | ***P*** | **MD（95%CI）** | ***P*** |
| B vs. A | 49.09（0.05,98.13） | 0.05 | 31.40（-21.20,84.00） | 0.27 |
| C vs. A | 25.56（-63.01,114.14） | 0.57 | 56.47（-39.79,152.73） | 0.47 |
| D vs. A | 19.54（-39.66,78.73） | 0.52 | -15.33（-89.06,58.39） | 0.30 |
| E vs. A | 39.99（-14.02,94.00） | 0.15 | 31.41（-21.25,84.07） | 0.27 |
| F vs. A | 69.39（9.54,129.24） | 0.02 | 100.29（27.51,173.07） | 0.07 |
| G vs. A | 29.39（-32.83,91.62） | 0.36 | 60.80（-14.60,136.20） | 0.81 |
| H vs. A | 23.04（-45.01,91.09） | 0.51 | 53.98（-25.11,133.07） | 0.70 |
| **overall inconsistency** | | | **x2= 1.78, *P* =0.18** | |

**Table S2. Inconsistency test between direct and indirect treatment comparisons in hepcidin**

| **node-splitting method** | | | | | | | | |
| --- | --- | --- | --- | --- | --- | --- | --- | --- |
| **Side** | **Direct** | | **Indirect** | | **Difference** | |  |  |
|  | **MD** | **SE** | **MD** | **SE** | **MD** | **SE** | ***P* >∣Z∣** |  |
| A B | 31.42 | 26.85 | 105.80 | 49.27 | -74.38 | 56.11 | 0.19 |  |
| A E | 31.42 | 26.85 | 180.17 | 109.18 | -148.75 | 112.22 | 0.19 |  |
| A F | 103.14 | 37.99 | 25.17 | 41.30 | 77.97 | 56.06 | 0.16 |  |
| B D | -46.72 | 26.41 | 28.29 | 49.76 | -75.01 | 56.34 | 0.18 |  |
| B E | 0.00 | 27.30 | -148.75 | 108.85 | 148.75 | 112.22 | 0.19 |  |
| C F | 43.82 | 33.32 | 115.13 | 5740.67 | -71.31 | 5740.79 | 0.99 |  |
| D F | 40.45 | 19.54 | 115.13 | 52.94 | -75.44 | 56.39 | 0.18 |  |
| F G | -39.99 | 12.71 | -129.56 | 430.15 | 89.56 | 430.14 | 0.84 |  |
| F H | -46.33 | 16.75 | -122.18 | 1197.41 | 75.84 | 1197.52 | 0.95 |  |
| **A: Daprodustat; B: Darbepoetin; C: Desidustat; D: Enarodustat; E: Epoetin; F: Placebo; G: Roxadustat; H: Vadadustat** | | | | | | | | |

| **overall inconsistency** | | | | |
| --- | --- | --- | --- | --- |
| **comparisons** | **Consistency Model** | | **inconsistency model** | |
|  | **MD（95%CI）** | ***P*** | **MD（95%CI）** | ***P*** |
| B vs. A | 0.40（-0.27,1.07） | 0.24 | 0.39（-0.47,1.24） | 0.38 |
| C vs. A | 1.16（0.08,2.24） | 0.04 | 1.15（-0.02,2.33） | 0.06 |
| D vs. A | 0.01（-0.69,0.72） | 0.97 | -0.02（-1.23,1.20） | 0.98 |
| E vs. A | 0.39（-0.38,1.17） | 0.32 | 0.39（-0.47,1.24） | 0.38 |
| F vs. A | -1.33（-1.91,-0.75） | 0.00 | -1.34（-2.02,-0.65） | 0.00 |
| G vs. A | 0.39（-0.28,1.06） | 0.26 | 0.38（-0.39,1.15） | 0.34 |
| H vs. A | -0.15（-0.89,0.59） | 0.69 | -0.16（-1.00,0.68） | 0.71 |
| **overall inconsistency** | | | **x2= 0.00, *P* =0.97** | |

**Table S3. Inconsistency test between direct and indirect treatment comparisons in Hb**

| **node-splitting method** | | | | | | | |
| --- | --- | --- | --- | --- | --- | --- | --- |
| **Side** | **Direct** | | **Indirect** | | **Difference** | |  |
|  | **MD** | **SE** | **MD** | **SE** | **MD** | **SE** | ***P* >∣Z∣** |
| A B | 0.39 | 0.44 | 0.41 | 0.65 | -0.03 | 0.77 | 0.97 |
| A E | 0.39 | 0.44 | 0.44 | 1.51 | -0.06 | 1.57 | 0.97 |
| A F | -1.34 | 0.35 | -1.36 | 0.70 | 0.02 | 0.78 | 0.97 |
| B D | -0.40 | 0.44 | -0.37 | 0.65 | -0.03 | 0.79 | 0.97 |
| B E | 0.00 | 0.44 | -0.06 | 1.51 | 0.06 | 1.57 | 0.97 |
| C F | -2.49 | 0.46 | -1.44 | 92.96 | -1.05 | 92.96 | 0.99 |
| D F | -1.35 | 0.33 | -1.32 | 0.71 | -0.03 | 0.77 | 0.97 |
| F G | 1.71 | 0.17 | 1.95 | 19.94 | -0.24 | 19.94 | 0.99 |
| F H | 1.17 | 0.24 | 2.08 | 15.01 | -0.91 | 15.01 | 0.95 |
| **A: Daprodustat; B: Darbepoetin; C: Desidustat; D: Enarodustat; E: Epoetin; F: Placebo; G: Roxadustat; H: Vadadustat** | | | | | | | |


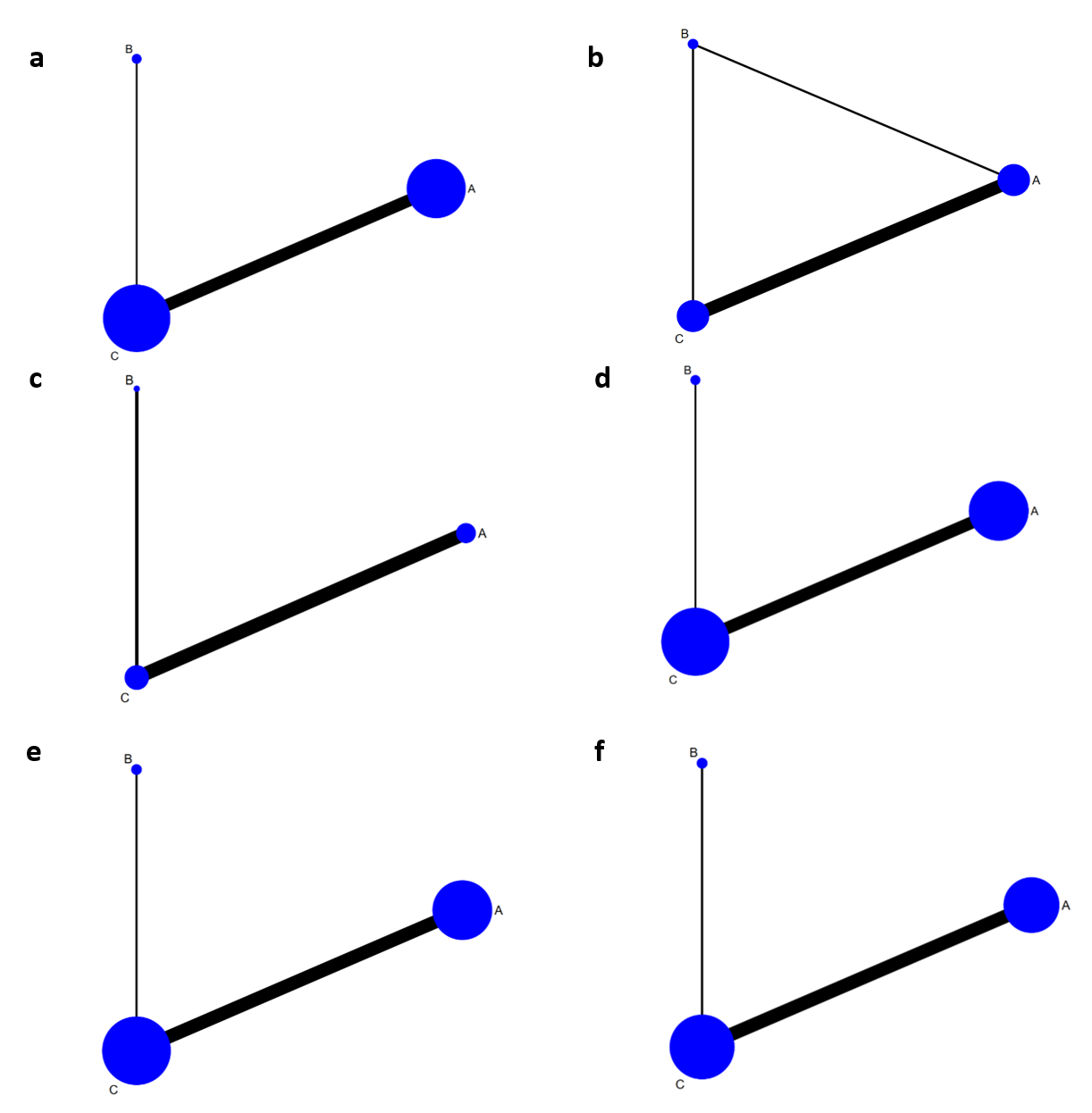


**Fig. S1.** Network graphs of all the drug agents included in the study. The width of the lines is proportional to the number of trials comparing each pair of treatments. The sizes of the circles are proportional to the number of trials using this intervention. A: placebo, B: ESAs, C: HIF-PHIs. (a): hepcidin, (b): serum iron, (c): transferrin, (d): ferritin, (e): TIBC, (f): TSAT.

PRISMA NMA Checklist

**PRISMA NMA Checklist of Items to Include When Reporting A Systematic Review Involving a Network Meta-analysis**

| **Section/Topic** | **Item #** | **Checklist Item** | **Reported on Page #** |
| --- | --- | --- | --- |
| **TITLE** | | |  |
| Title | 1 | Identify the report as a systematic review *incorporating a*  *network meta-analysis (or related form of meta-analysis).* | 1 |
| **ABSTRACT** |  |  | 2 |
| Structured summary | 2 | Provide a structured summary including, as applicable:  **Background:** main objectives  **Methods:** data sources; study eligibility criteria, participants, and interventions; study appraisal; and *synthesis methods, such as network meta-analysis.*  **Results:** number of studies and participants identified; summary estimates with corresponding confidence/credible intervals; *treatment rankings may also be discussed. Authors may choose to summarize pairwise comparisons against a chosen treatment included in their analyses for brevity.*  **Discussion/Conclusions:** limitations; conclusions and implications of findings.  **Other:** primary source of funding; systematic review registration number with registry name. |  |
| **INTRODUCTION** |  |  |  |
| Rationale | 3 | Describe the rationale for the review in the context of what is  already known*, including mention of why a network meta- analysis has been conducted.* | 2-3 |
| Objectives | 4 | Provide an explicit statement of questions being addressed, with reference to participants, interventions, comparisons, outcomes, and study design (PICOS). | 2-3 |
| **METHODS** |  |  |  |
| Protocol and registration | 5 | Indicate whether a review protocol exists and if and where it can be accessed (e.g., Web address); and, if available, provide  registration information, including registration number. | 3 |
| Eligibility criteria | 6 | Specify study characteristics (e.g., PICOS, length of follow-up) and report characteristics (e.g., years considered, language, publication status) used as criteria for eligibility, giving rationale. *Clearly describe eligible treatments included in the treatment network, and note whether any have been clustered*  *or merged into the same node (with justification).* | 3-4 |
| Information sources | 7 | Describe all information sources (e.g., databases with dates of  coverage, contact with study authors to identify additional studies) in the search and date last searched. | 4 |
| Search | 8 | Present full electronic search strategy for at least one database,  including any limits used, such that it could be repeated. | 4  Fig. 1 |
| Study selection | 9 | State the process for selecting studies (i.e., screening, eligibility, included in systematic review, and, if applicable, | 3-4 |

|  |  | included in the meta-analysis). |  |
| --- | --- | --- | --- |
| Data collection  process | 10 | Describe method of data extraction from reports (e.g., piloted  forms, independently, in duplicate) and any processes for  obtaining and confirming data from investigators. | 3 |
| Data items | 11 | List and define all variables for which data were sought (e.g.,  PICOS, funding sources) and any assumptions and  simplifications made. | 3-4 |
| **Geometry of the**  **network** | **S1** | Describe methods used to explore the geometry of the  treatment network under study and potential biases related to it.  This should include how the evidence base has been  graphically summarized for presentation, and what  characteristics were compiled and used to describe the evidence  base to readers. | Fig. 4  Fig. S1 |
| Risk of bias within  individual studies | 12 | Describe methods used for assessing risk of bias of individual  studies (including specification of whether this was done at the  study or outcome level), and how this information is to be used  in any data synthesis. | 4 |
| Summary measures | 13 | State the principal summary measures (e.g., risk ratio,  difference in means). *Also describe the use of additional*  *summary measures assessed, such as treatment rankings and*  *surface under the cumulative ranking curve (SUCRA) values,*  *as well as modified approaches used to present summary*  *findings from meta-analyses.* | 3-4 |
| Planned methods of  analysis | 14 | Describe the methods of handling data and combining results of  studies for each network meta-analysis. This should include,  but not be limited to:   - *Handling of multi-arm trials;* - *Selection of variance structure;* - *Selection of prior distributions in Bayesian analyses;*   *and*   - *Assessment of model fit.* | 4 |
| **Assessment of**  **Inconsistency** | **S2** | Describe the statistical methods used to evaluate the agreement  of direct and indirect evidence in the treatment network(s)  studied. Describe efforts taken to address its presence when  found. | 4-5 |
| Risk of bias across  studies | 15 | Specify any assessment of risk of bias that may affect the  cumulative evidence (e.g., publication bias, selective reporting  within studies). | 4 |
| Additional analyses | 16 | Describe methods of additional analyses if done, indicating  which were pre-specified. This may include, but not be limited  to, the following: | - |
| - Sensitivity or subgroup analyses; - Meta-regression analyses; - *Alternative formulations of the treatment network; and* - *Use of alternative prior distributions for Bayesian analyses (if applicable).* | | | |

| **RESULTS** |  |  | |
| --- | --- | --- | --- |
| Study selection | 17 | Give numbers of studies screened, assessed for eligibility, and included in the review, with reasons for exclusions at each stage, ideally with a flow diagram. | 5  Fig. 2 |
| **Presentation of**  **network structure** | **S3** | Provide a network graph of the included studies to enable  visualization of the geometry of the treatment network. | Fig. 4  Fig. S1. |
| **Summary of network geometry** | **S4** | Provide a brief overview of characteristics of the treatment network. This may include commentary on the abundance of trials and randomized patients for the different interventions and pairwise comparisons in the network, gaps of evidence in the treatment network, and potential biases reflected by the network structure. | 5 |
| Study characteristics | 18 | For each study, present characteristics for which data were extracted (e.g., study size, PICOS, follow-up period) and  provide the citations. | 5  Table.1  Table S1 |
| Risk of bias within studies | 19 | Present data on risk of bias of each study and, if available, any outcome level assessment. | 5  Fig. 3 |
| Results of individual studies | 20 | For all outcomes considered (benefits or harms), present, for each study: 1) simple summary data for each intervention group, and 2) effect estimates and confidence intervals.  *Modified approaches may be needed to deal with information from larger networks.* | *5-6* |
| Synthesis of results | 21 | Present results of each meta-analysis done, including confidence/credible intervals. *In larger networks, authors may focus on comparisons versus a particular comparator (e.g. placebo or standard care), with full findings presented in an appendix. League tables and forest plots may be considered to summarize pairwise comparisons.* If additional summary measures were explored (such as treatment rankings), these  should also be presented. | 5-6 |
| **Exploration for inconsistency** | **S5** | Describe results from investigations of inconsistency. This may include such information as measures of model fit to compare consistency and inconsistency models, *P* values from statistical tests, or summary of inconsistency estimates from different  parts of the treatment network. | 5  Table S2-S3 |
| Risk of bias across studies | 22 | Present results of any assessment of risk of bias across studies for the evidence base being studied. | 5-7  Fig. 3  Fig. 8 |
| Results of additional analyses | 23 | Give results of additional analyses, if done (e.g., sensitivity or subgroup analyses, meta-regression analyses*, alternative network geometries studied, alternative choice of prior distributions for Bayesian analyses,* and so forth). | - |
| **DISCUSSION** |  |  | |
| Summary of evidence | 24 | Summarize the main findings, including the strength of evidence for each main outcome; consider their relevance to key groups (e.g., healthcare providers, users, and policy-  makers). | 7 |
| Limitations | 25 | Discuss limitations at study and outcome level (e.g., risk of bias), and at review level (e.g., incomplete retrieval of identified research, reporting bias). *Comment on the validity of*  *the assumptions, such as transitivity and consistency. Comment* | *9* |

| *on any concerns regarding network geometry (e.g., avoidance of certain comparisons).* | | |  |
| --- | --- | --- | --- |
| Conclusions | 26 | Provide a general interpretation of the results in the context of other evidence, and implications for future research. | 7-9 |
| **FUNDING** |  |  | 9 |
| Funding | 27 | Describe sources of funding for the systematic review and other  support (e.g., supply of data); role of funders for the systematic review. This should also include information regarding whether funding has been received from manufacturers of treatments in the network and/or whether some of the authors are content experts with professional conflicts of interest that could affect  use of treatments in the network. |  |

PICOS = population, intervention, comparators, outcomes, study design.

* Text in italics indicateS wording specific to reporting of network meta-analyses that has been added to guidance from the PRISMA statement.

† Authors may wish to plan for use of appendices to present all relevant information in full detail for items in this section.
